# Increased default mode network connectivity and increased regional homogeneity in migraineurs without aura

**DOI:** 10.1186/s10194-016-0692-z

**Published:** 2016-10-22

**Authors:** Jilei Zhang, Jingjing Su, Mengxing Wang, Ying Zhao, Qian Yao, Qiting Zhang, Haifeng Lu, Hui Zhang, Shuo Wang, Ge-Fei Li, Yi-Lan Wu, Feng-Di Liu, Yan-Hui Shi, Jianqi Li, Jian-Ren Liu, Xiaoxia Du

**Affiliations:** 1 Shanghai Key Laboratory of Magnetic Resonance and Department of Physics, East China Normal University, 3663 North Zhong-Shan Road, Shanghai, 200062 People’s Republic of China; 2 Department of Neurology and Jiuyuan Municipal Stroke Center, Shanghai Ninth People’s Hospital, Shanghai Jiao Tong University School of Medicine; and Clinical Research Center, Shanghai Jiao Tong University School of Medicine, 639 Zhizaoju Road, Shanghai, 200011 People’s Republic of China

**Keywords:** Migraine, Precuneus, Functional connectivity, Regional homogeneity

## Abstract

**Background:**

The precuneus/posterior cingulate cortex, which has been associated with pain sensitivity, plays a pivotal role in the default mode network. However, information regarding migraine-related alterations in resting-state brain functional connectivity in the default mode network and in local regional spontaneous neuronal activity is not adequate.

**Methods:**

This study used functional magnetic resonance imaging to acquire resting-state scans in 22 migraineurs without aura and in 22 healthy matched controls. Independent component analysis, a data-driven method, was used to calculate the resting-state functional connectivity of the default mode network in the patient and healthy control groups. Regional homogeneity (ReHo) was used to analyse the local features of spontaneous resting-state brain activity in the migraineurs without aura.

**Results:**

Compared with the healthy controls, migraineurs without aura showed increased functional connectivity in the left precuneus/posterior cingulate cortex within the default mode network and significant increase in ReHo values in the bilateral precuneus/posterior cingulate cortex, left pons and trigeminal nerve entry zone. In addition, functional connectivity was decreased between the areas with abnormal ReHo (using the peaks in the precuneus/posterior cingulate cortex) and other brain areas.

**Conclusions:**

The abnormalities in the precuneus/posterior cingulate cortex suggest that migraineurs without aura may exhibit information transfer and multimodal integration dysfunction and that pain sensitivity and pian processing may also be affected.

## Background

In the past decade, neuroimaging studies have provided important new insight into migraine. Previous task-evoked functional magnetic resonance imaging (fMRI) studies consistently showed that migraines are associated with atypical brain activation in response to painful, olfactory and visual stimuli [1, 2]. Resting-state functional connectivity (FC) fMRI studies have identified numerous brain regions and functional networks with atypical FC in migraineurs, suggesting that migraines are associated with aberrant brain functional organization [1, 3–5]. Several analytical techniques have been used in FC analyses of migraine [3], such as seed-based analysis, independent component analysis (ICA), regional homogeneity (ReHo) and graph theory approaches. These studies have significantly contributed to better understanding of the mechanisms of migraine from different points of view.

ICA can be used to separate the spatiotemporal component from whole-brain fMRI data, and the grouping of brain activity provided by each component reflects regions with the same response pattern, providing a natural measure of FC. The ReHo method tests for local correlations in blood oxygen level-dependent (BOLD) time series using Kendall’s coefficient of concordance (KCC) to measure regional synchronizations of temporal changes in BOLD activity in a voxel-wise manner; ReHo thus reflects the temporal synchrony of the BOLD signal [6]. ReHo and ICA are data-driven approaches and thus require no prior knowledge. ReHo analysis and ICA have been used to study depression, migraine [7–11] and other neurological disorders.

The default mode network (DMN), which includes the precuneus, posterior cingulate cortex (PCC), medial prefrontal cortex, medial temporal lobe and angular gyrus, is one of the main networks that are consistently identified when an individual is at wakeful rest and not performing an attention-demanding task. One task-based fMRI study observed that some DMN subregions responded in a percept-related manner to pain, suggesting closer link between the DMN and pain processing than previously thought [12]. Previous studies have also been found greater connectivity between the DMN and the executive network and the insular cortex [13], and abnormal connectivity within the DMN [3, 11, 14], the results were not consistent. The precuneus/PCC plays a pivotal role in the DMN [15], and the precuneus is associated with pain sensitivity [16–18]. Goffaux et al. found that large pain-related increases in precuneus activity were associated with small increments in the sural nerve stimulation strength required to progress from a non-painful to a painful state [16]. Previous studies also showed that migraineurs were hypersensitive to somatosensory, visual, auditory and olfactory stimuli, exhibiting lower discomfort thresholds [1, 2, 19]. Thus, we predicted that migraineurs without aura would exist abnormal connectivity in the precuneus/PCC of the DMN, which may be associated with pain hypersensitive and abnormal pain processing in migraineurs.

Five studies from two groups have used the ReHo method to investigate the local features of spontaneous brain activity in patients with migraine. Yu et al. reported significant decreases in ReHo values in the right rostral anterior cingulate cortex, the prefrontal cortex, the orbitofrontal cortex and the supplementary motor area [8] and they also observed that the ReHo patterns in migraine patients were also affected by depressive symptoms [9]. Zhao et al. found that the increased average ReHo values in the thalamus, brain stem and temporal pole were significantly positively correlated with the disease duration [7]. Zhao et al. also used the ReHo method to investigate the effectiveness of acupuncture therapy and to characterize longitudinal changes in brain activity in a group of female migraine patients [10]. Although, previous studies have found abnormal ReHo values in migraine patiens; the results have been inconsistent, and the functional connectivity between the ReHo abnormal areas and the other parts of the brain remains unclear.

We hypothesized that migraineurs without aura would exist atypical connectivity in the precuneus/PCC of the DMN, and the local ReHo would be disrupted too. In this study ICA analysis was to investigate DMN connectivity in patients with migraine under resting-state. In addition, ReHo analysis was used to identify the local features of spontaneous brain activity in the migraine patients, and seed-based FC was used to investigate how the ReHo abnormalities areas affect the rest of the brain.

## Methods

The study was approved by the East China Normal University Committee on Human Research (Project No. HR2015/03011) and by the Independent Ethics Committee of Shanghai Ninth People’s Hospital (Project No. [2016]01). All subjects gave written informed consent using forms approved by the committee.

### Subjects

Twenty-two migraineurs without aura (9 males, 13 females) were recruited from among outpatients of the Department of Neurology at Shanghai Ninth People’s Hospital. These patients were diagnosed as migraineurs without aura by a neurologist according to the International Classification of Headache Disorders (ICHD-II, 2004) [20]. The neurologist also obtained the patients’ demographic and clinical data, including age, sex, disease duration, attack frequency (times/month) and attack duration as well as visual analogue scale (VAS), Migraine Disability Assessment Scale (MIDAS) and Headache Impact Test (HIT-6) scores. The migraineurs without aura all reported that they did not suffer migraine attack or discomfort during the MRI scans. Twenty-two age- and gender-matched healthy controls (9 males, 13 females) were recruited if they had not experienced any headaches in the past year and if their family members did not suffer from migraine or other headaches. All subjects were right handed and had no substance abuse, and all neurological and psychiatric diseases were excluded based on clinical examination and a structured interview. To assess the participants’ depression and anxiety state, all subjects were required to complete the 24-Hamilton Depression Rating Scale (24-HAMD) and 14-Hamilton Anxiety Scale (14-HAMA). Details are provided in Table 1.

**Table 1:** Demographics and clinical scores of the migraineurs without aura and the controls

	Migraine group (Mean ± SD)	Control group (Mean ± SD)
Male/Female	9/13	9/13
Age (years)	41.8 ± 10.2	42.0 ± 10.3
Disease duration (years)	9.8 ± 7.3	-
Attack duration (hours)	18.0 ± 15.5	
Attackfrequency (times/months)	3.1 ± 2.2	
VAS	7.7 ± 1.9	-
MIDAS	10.4 ± 7.6	-
HIT-6	61.2 ± 9.6	-
HAMA	4.4 ± 4.5	1.2 ± 0.7**
HAMD	3.0 ± 2.6	1.7 ± 0.8*

*MIDAS* migraine disability assessment scale, *HIT-6* headache impact test, *HAMA* Hamilton anxiety scale, *HAMD* Hamilton depression scale

*-* no data

*P < 0.1 migraine subjects compared with control subjects

**P < 0.001 migraine subjects compared with control subjects

### MRI acquisition

Functional and structural MRI data were acquired using a 3.0 Tesla Siemens Trio Tim system that utilized a 12-channel head coil. All subjects’ head movements were minimized with custom-fit foam pads. We obtained the whole-brain anatomical volume using a high-resolution T_1_-weighted 3-dimensional magnetization-prepared rapid-acquisition gradient-echo pulse sequence. The parameters were as follows: repetition time = 2530 ms, echo time = 2.34 ms, inversion time = 1100 ms, flip angle = 7°, number of slices = 192, sagittal orientation, field of view = 256 × 256 mm^2^, matrix size = 256 × 256, and slice thickness = 1 mm, 50 % gap. The resting-state fMRI images were acquired using a T_2_*-weighted gradient-echo echo-planar imaging pulse sequence with the following parameters: repetition time = 2000 ms, echo time = 30 ms, flip angle = 90°, number of slices = 32, transverse orientation, field of view = 220 × 220 mm^2^, matrix size = 64 × 64, slice thickness = 3.5 mm, 25 % Dist factor, and total volumes = 210. During the fMRI scan, the subjects were instructed to relax, hold still and close their eyes.

### Resting-state fMRI data preprocessing

Resting-state fMRI data preprocessing was conducted with statistical parametric mapping software (SPM12; http://www.fil.ion.ucl.ac.uk/spm/software/spm12) and MATLAB (The Math Works, Natick, MA) software on a personal computer. For each participant, the first 10 volumes were discarded to avoid scanner instability and to adapt the participants to the noise of the scanner. The remaining volumes were corrected for the intra-volume acquisition time delay using slice-timing and were realigned to the first volume using the six-parameter (rigid body) spatial transformation. After these corrections, we co-registered the high-resolution T_1_-weighted image to the mean functional image. The T_1_ images were then segmented into grey matter, white matter and bias field-corrected structural images. Then, the images were spatially normalized to the standard Montreal Neurological Institute (MNI) stereotaxic space and resampled to 3 × 3 × 3 mm. Finally, spatial smoothing was performed on the functional images using a Gaussian filter (8 mm full width half-maximum, FWHM).

The head motion parameters of all participants were calculated in the translational and rotational directions (i.e., x, y, z, roll, pitch and yaw). Participants were excluded if their maximum translation was > 2 mm or their rotation was > 2° in any direction; none of the participants exhibited excessive movement. Head motion in all directions was compared between the groups, and we found that the head motion parameters of the migraine and control groups did not significantly differ (*x*, *P* = 0.79; *y*, *P* = 0.99; *z*, *P* = 0.82; pitch, *P* = 0.62; *roll*, *P* = 0.70; yaw, *P* = 0.99.).

### Data analysis

#### ICA and DMN analysis

We applied ICA to the preprocessed data using Group ICA of the fMRI Toolbox (GIFT 4.0a, http://icatb.sourceforge.net/), which utilizes the Infomax algorithm. The preprocessed group data were decomposed into 20 spatial ICs (the default value). The data were concatenated and reduced by two-stage principal component analysis (PCA). Subsequently, the ICs were calculated using the Infomax algorithm. The GICA-3 back reconstruction step was used to separate single-subject components from the set of aggregate components calculated by the previous step. Finally, for all subjects, spatial components maps were acquired and converted to z-score maps.

The DMN was selected by visual inspection from 20 aggregate spatial ICs and based on brain regions associated with the DMN in previous studies [21, 22]. To estimate intra-group spatial consistency, we performed one-sample t-tests (*P* < 0.001, false discovery rate (FDR) corrected) on the DMN spatial maps for migraineurs without aura group and the control group. Two-sample t-tests were used to evaluate inter-group differences within a mask created by the union map of the one-sample t-test images, and the covariates age and gender were removed to control their effects. To address the issue of multiple comparisons, the statistical maps were thresholded at *P* < 0.001 (voxel level) and FWE corrected to *P* < 0.05 at the cluster level. The surviving clusters were reported.

#### ReHo and FC analysis

We used DPABI v2.1 to conduct the ReHo and FC analysis. Linear trends were removed from the unsmoothed data. Spurious signals, including the time series of six head motion parameters, the white matter and the cerebrospinal fluid were regressed out using a general linear model based on the fMRI data. Then, a temporal band-pass filter (0.01 < f < 0.1 Hz) was applied to reduce the influence of low-frequency drift and high-frequency respiratory and cardiac noise.

An individual ReHo map was generated by calculating the concordance of KCC of the time series of a given voxel with those of its 26 nearest neighbours [6]. To eliminate the effect of individual diversification, the ReHo value of each voxel was converted into a z-score by subtracting the mean ReHo value and dividing by the standard deviationof the whole-brain ReHo map. The standardized ReHo maps were spatially smoothed withan 8 mm FWHM Gaussian kernel. Before evaluating FC, we smoothed the filtered fMRI data with an 8 mm FWHM Gaussian kernel. The brain regions for which ReHo was significantly altered were used as seed regions in the whole-brain FC analysis. Then, we extracted the mean time course from the seed regions (using a 6-mm spherical region of interest (ROI) centred at the peak significant coordinate), and Pearson correlation was used to correlate these time courses with whole-brain voxels. Finally, the FC maps were converted to z-score maps by Fisher Z-transformation.

The maps of significant differences in ReHo and FC maps of the 22 migraineurs without aura and the 22 age- and gender-matched controls were compared using voxel-wise two-sample t-tests with age and gender as covariates within a brain mask. The ReHo statistical maps used the same correction method as the ICA statistical analysis to address the issue of multiple comparisons. A stricter correction level (*p* < 0.005, FDR corrected) was used for the FC statistical maps because we obtained too many clusters if we used the same multiple comparison correction methods described above. The surviving clusters were reported.

#### Correlation analysis

Individual mean ICA z-scores and ReHo z-scores for the surviving clusters of the migraineurs without aura and control groups were extracted for linear correlation with the clinical data, including disease duration, attack frequency and attack duration, as well as VAS, MIDAS, HIT-6, HAMA and HADA scores.

## Results

### Demographic and clinical data of the migraineurs without aura and control groups

The demographic and clinical data of migraineurs without aura and controls are presented in Table 1. The age and gender demographic factors did not significantly differ between the migraineurs without aura and control groups. In addition, although anxiety and depression disorders were excluded in our study, we found significantly increased HAMD scores (*P* < 0.001) and slight increased HAMA scores (*P* < 0. 1) in the migraineurs without aura group (see Table 1).

### ICA

**Fig. 1:**
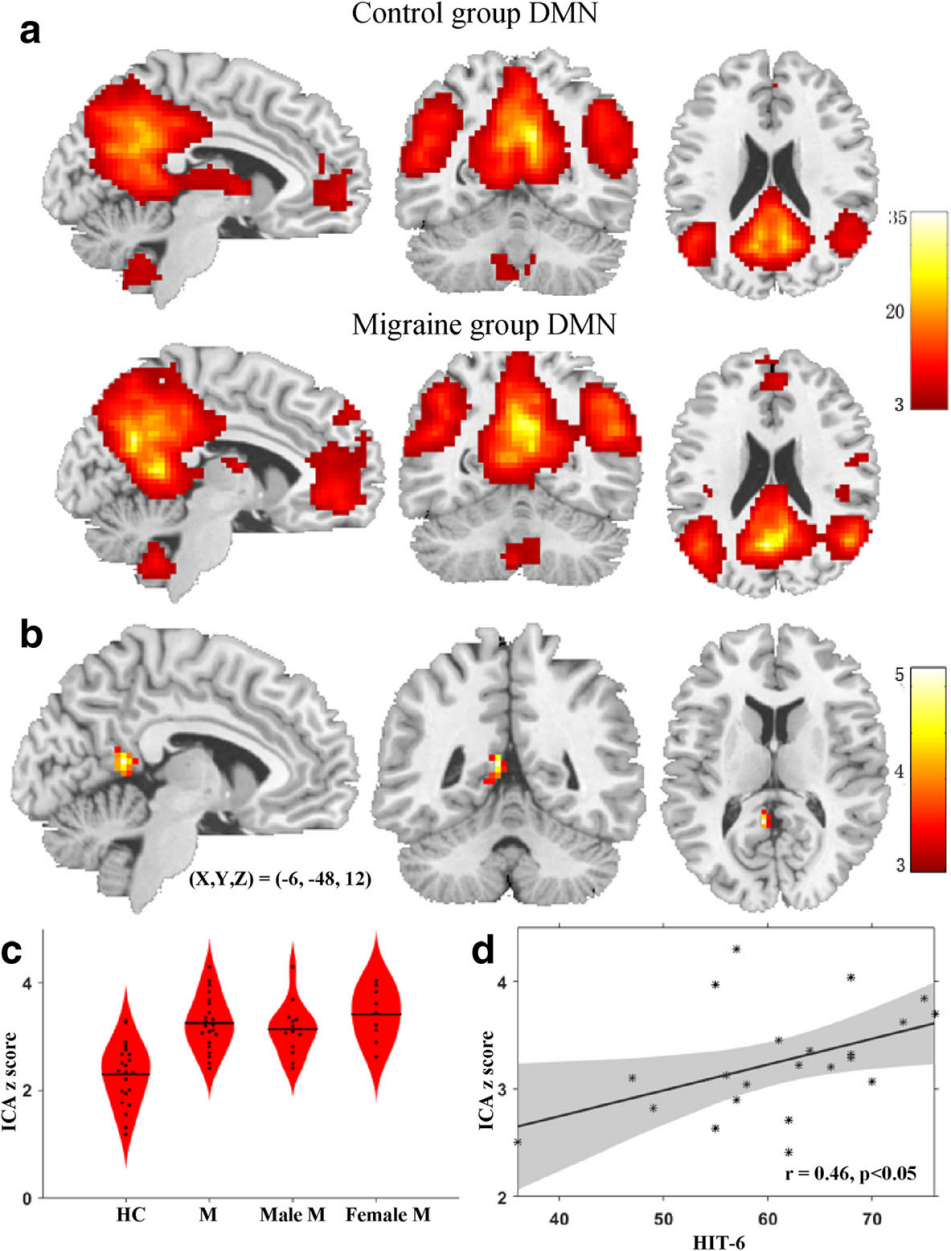
The default mode network (DMN) functional connectivity in migraineurs without aura and healthy controls and the differences between the groups. **a** DMN network of each group was obtained by one-sample *t* test (FDR corrected, *p* = 0.001). The brain areas of the DMN comprised of the medial prefrontal cortex, anterior and posterior cingulate cortexes, precuneus, angular gyrus and cerebellar areas in the migraine and control groups. **b** Compared with the control group, the migraineurs without aura showed significantly increased intra-network connectivity in the left posterior cingulate and precuneus. **c** The mean ICA z-scores were extracted from the significantly altered brain area in each subject. No significant differences in intra-network FC were observed between the female and male migraineurs. **d** The ICA z-score of the precuneus/PCC was positively correlated with the HIT-6 score (*r* = 0.46, *P* < 0.05). HC, healthy control; M, migraineurs without aura

The DMN connectivity pattern of the migraineurs without aura and control groups was consistent with those found in previous studies, significant differences could not be identified by visual inspection (Fig. 1). The brain areas in the DMN included the medial prefrontal cortex, anterior and posterior cingulate cortices, precuneus, angular gyrus and cerebellar areas. Compared with the control group, the migraineurs without aura showed increased intra-network connectivity in the left PCC and precuneus (Table 2 and Fig. 1b).

**Table 2:** Group differences in default mode network functional connectivity and regional homogeneity between migraineurs without aura and healthy controls

Predominant regions in cluster	Cluster size	PeakT value	MNI coordinates			Cluster-level
			x	y	z	P_FWE-corr_
DMN functional connectivity increase in migraine group						
Left posterior cingulate cortex and precuneus	30	4.83	−6	−48	12	0.070
ReHo increase in migraine group						
Bilateral posterior cingulate cortex and precuneus	204	5.66	−6	−54	18	0.001
Left pons and trigeminal nerve entry zone	115	5.40	−18	−21	−36	0.014

*P* < 0.001 at voxel level and alteration significant at the clusterlevel (*P <* 0.05, FWE corrected) will be reported

### ReHo and FC

**Fig. 2:**
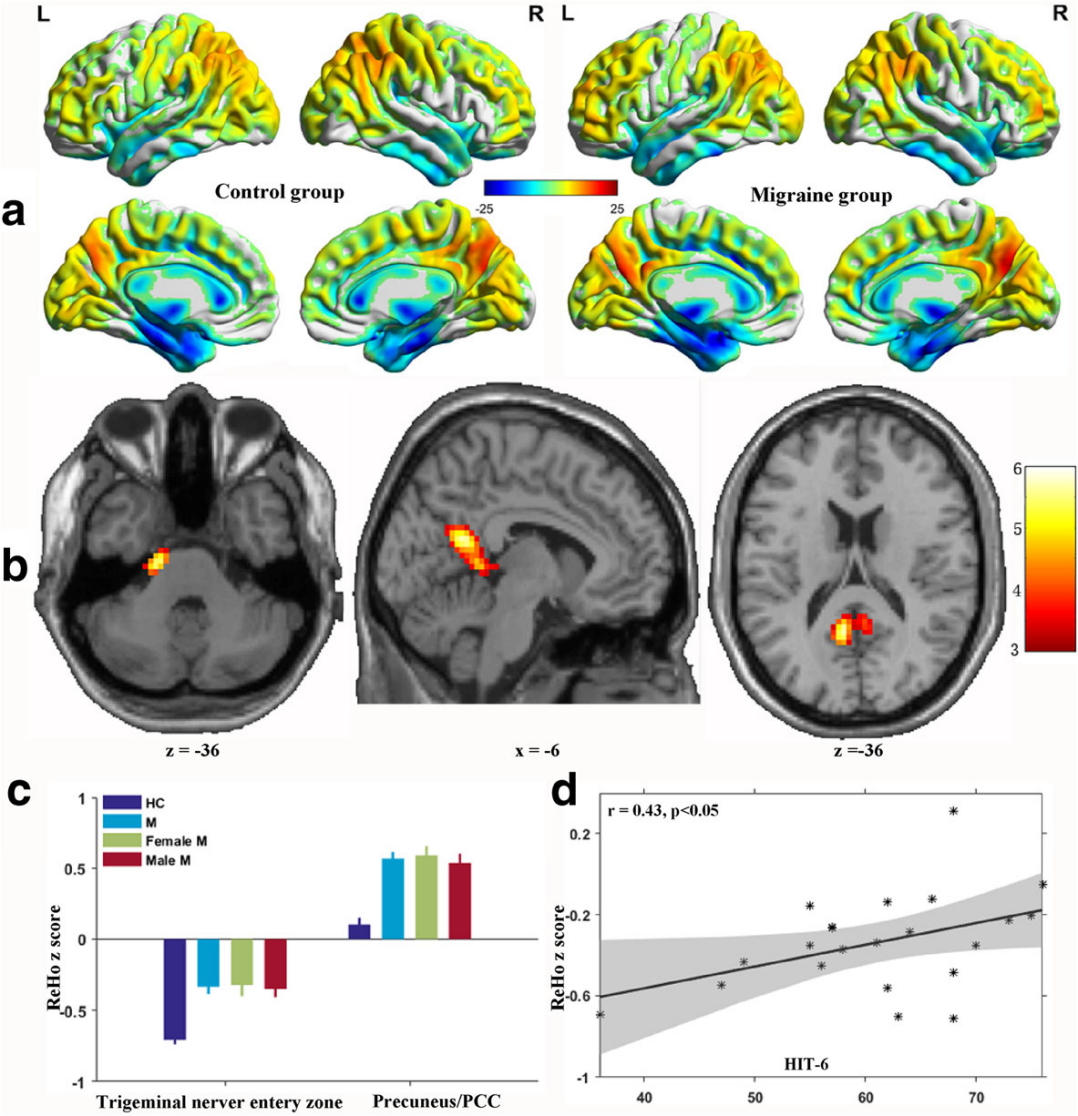
Regional homogeneity (ReHo) maps in migraineurs without aura and healthy controls and the differences between groups. **a** The ReHo maps of each group were obtained by one-sample *t* test (FDR corrected, *P* = 0.001). **b** When comparing controls, the migraine patients exhibited significantly increased ReHo values in the bilateral precuneus/PCC, pons and trigeminal nerver entery zone. **c** The mean ReHo z-scores were extracted within the significantly altered brain area of each subject. There were no significant differences in ReHo between the female and male migraineurs without aura. **d** The ReHo z-score of the trigeminal nerver entery zone was positively correlated with the HIT-6 score (*r* = 0.43, *p* < 0.05). HC, healthy control; M, migraineurs without aura

Compared with the controls, the migraineurs without aura exhibited significantly increased ReHo values in the bilateral precuneus and PCC, left pons and trigeminal nerve entry zone (Table 2 and Fig. 2b). To further investigate the FC between the areas of abnormal ReHo and other brain areas, we compared the FC between the precuneus and other brain regions between the migraineurs without aura and the controls using the peak in the precuneus/PCC as the ROI (region of interest) seed. The FC of the precuneus with the whole-brain was compared between the migraineurs without aura and the age- and gender-matched controls. The migraineurs without aura showed weaker FC between the left precuneus and the left inferior and superior occipital gyri, bilateral middle occipital gyri, bilateral cuneus, bilateral superior parietal lobules, bilateral somatosensory cortex (postcentral gyrus), right dorsolateral prefrontal cortices (middle, superior and inferior frontal gyri), left dorsolateral prefrontal cortices (middle and inferior frontal gyri), right premotor cortex, pons, bilateral cerebellar posterior lobes, right paracentral lobule, right middle cingulate gyrus and bilateral supplementary motor areas (Table 3).

**Table 3:** Significant inter-group differences identified in the ReHo-seed (precuneus) functional analysis

Predominant regions in cluster	Cluster size	PeakT value	MNI coordinates		
			x	y	z
Migraine group < Control group					
Left inferior occipital gyrus	202 (3862)	6.34	−42	−72	−9
Bilateral middle occipital gyri	545 (3862)	5.23	−42	−78	6
		5.10	45	−54	6
Left superior occipital gyrus	215 (3862)	4.71	21	−66	42
Bilateral cuneus	924 (3862)	5.32	−12	−93	21
		5.24	18	−84	39
Bilateral superior parietal lobules	332 (3862)	5.03	−18	−51	57
		4.68	12	−63	66
Left postcentral gyrus	165 (3862)	4.59	−24	−42	66
Right middle and superior frontal gyri (BA10 and BA 9)	375	6.27	21	57	27
Left middle frontal gyrus	117	4.30	−30	48	24
Bilateral cerebellum posterior lobes	441	4.64	−18	−78	−54
Right inferior frontal gyrus (BA47)	185	5.57	39	24	−18
Left inferior frontal gyrus	100	5.39	−45	18	3
Right postcentral gyrus (BA3 and BA2)	177	5.37	60	−24	42
Right middle frontal cortex (BA6)	227	5.02	27	−9	63
Pons	83	4.81	9	−30	−27
Right paracentral lobule	66	4.69	15	−36	45
Right middle cingulate gyrus	62	4.48	12	24	30
Bilateral supplementary motor areas	126	4.29	−9	12	45

Alteration significant brain areas at the voxel level correction (*P <* 0.005, FDR corrected, cluster size > 50 voxels) will be reported

### Correlation with clinical scores

We found that the ICA z-score of the precuneus/PCC and the ReHo z-score of the trigeminal nerver entery zone were positively correlated with the HIT-6 score (Figs. 1d and 2d). There were no significant correlation between the mean ReHo/ICAz-scores within the altered brain areas and disease duration, attack frequency and attack duration, as well as VAS, MIDAS, HAMA and HADA scores in the migraineurs without aura.

## Discussion

The main study finding is that the FC in the bilateral precuneus/PCC within the DMN was increased; ReHo values were also increased in the bilateral precuneus/PCC, and the FC between the precuneus and other brain areas was decreased. These results suggest that the inter-FC within the precuneus/PCC was increased, while the FC with other brain areas was decreased. Our data demonstrate precuneus/PCC dysfunction in migraineurs without aura.

High DMN activity occurs when the mind is not engaged in specific behavioural tasks; in contrast, DMN activity is low when attention is focused on the external environment, and the DMN has been implicated in mind wandering, self- referential processing, spontaneous cognition and consciousness [23]. Lower FC in the DMN has been observed in neuropsychiatric conditions such as Alzheimer’s disease, minimally conscious states and vegetative states [24, 25]. The precuneus/PCC, which is the strongest hub in the brain has been functionally linked to regions that constitute the DMN and plays a pivotal role in the DMN [15, 26]. The precuneus/PCC hub has been proposed to participate in information transfer and multimodal integration, which might be essential for the processing of spontaneous thoughts and for internal awareness [26]. Previous task-based fMRI studies have reported greater visual stimuli-induced precuneus activation in migraineurs without aura than in controls [1]. Kim et al. also reported that migraineurs without aura exhibited significant hypometabolism in the PCC compared with a healthy group [27]. The high FC and ReHo values of the precuneus/PCC indicate an increase in the correlations and synchronizations of local spontaneous brain activity, suggesting abnormal functional of the precuneus/PCC. Furthermore, the value of FC for the precuneus/PCC within DMN was positively correlated with the HIT-6 score, which suggests that the abnormally high FC strength of the precuneus/PCC would plays an role in the pathophysiology of migraine and would be impact the patient’s daily of life.

Goffaux et al. found that pain sensitivity in healthy adults was closely tied to pain-evoked responses in the contralateral precuneus [16]. In addition, Emerson at al. investigated the relationship between grey matter density and interindividual differences in pain sensitivity in 116 healthy volunteers and revealed a significant inverse relationship between pain sensitivity and grey matter density in bilateral regions of the PCC, precuneus, intraparietal sulcus and inferior parietal lobule [17]. Schwedt et al. also found negative correlations between pain thresholds and cortical thickness in the left precuneus/PCC in healthy controls; by contrast, migraineurs without aura exhibited positive correlations between pain thresholds and cortical thickness in the right precuneus [18]. In our study, the migraineurs without aura showed abnormal spontaneous brain activity in the bilateral precuneus/posterior cingulate cortex.

Compared with the controls, the migraineurs without aura showed a significant decrease in functional resting-state connectivity between the left precuneus and the left inferior and superior occipital gyri, bilateral middle occipital gyri, bilateral cuneus, bilateral superior parietal lobules, bilateral somatosensory cortex, bilateral dorsolateral prefrontal cortices, right premotor cortex, pons, bilateral cerebellar posterior lobes, right paracentral lobule, right middle cingulate gyrus and bilateral supplementary motor areas. These brain regions are all involved in the processing of pain [1]. The bilateral somatosensory cortex, left premotor cortex, bilateral dorsolateral prefrontal cortices, right premotor cortex, bilateral supplementary motor areas and bilateral superior parietal lobules participate in the discrimination of sensory features of pain [28]. Thus, overall, the migraineurs without aura showed increased FC and ReHo values within the precuneus/PCC and decreased FC with other brain areas; these observations suggest precuneus/PCC dysfunction, including local overactivity and abnormal linkage with other brain areas. The ventral precuneus/PCC is one of the major cortical hubs were linked to 4 cortical networks: the default mode, dorsal attention, visual and somatosensory networks [26]. The precuneus/PCC is considered the central node of the DMN and is involved in information transfer and multimodal integration [26, 29]. In a recent review, PCC function was proposed to included three dimensions (i) the state of arousal; (ii) the balance between internally and externally focused attention; and (iii) the breadth of attention [30]. The precuneus is also associated with pain sensitivity [16–18]. Thus, the abnormalities in the precuneus/PCC, which suggest that migraineurs without aura may suffer from information transfer and multimodal integration dysfunction as well as a high arousal state, imbalance between internally and externally directed attention and multisensory integration abnormalities, may also result in hypersensitivity to somatosensory, visual, auditory and olfactory stimuli and affect pain sensitivity and processing [1, 19, 26, 29, 30].

We also observed increased ReHo in the pons and trigeminal nerve entry zone (which are agreeable with the principal trigeminal nuclei) in migraineurs without aura. These areas are all involved in pain processing. The pons and trigeminal nerve entry zone are all part of the trigeminovascular pathway, which conveys nociceptive information from the meninges to the brain [31]. Previous studies supports the notion that the brainstem has an important role in the complex pathophysiology of migraine headache and suggest a possible role of the dorsolateral pons in generating migraine attacks [32, 33]. The increased ReHo in these areas may reflect damage to modulatory systems that then cannot inhibit nociceptive drive from trigeminovascular afferents [34]. We observed that the ReHo properties of the trigeminal nerver entery zone were positively correlated with the HIT-6 score, which suggests that the abnormal degree of ReHo in the trigeminal nerve during the resting-state would affect the paitient’s quality of life.

Although our research is informative with respect to the abnormal FC in the precuneus/PCC within the DMN as well as the increased ReHo value in the precuneus/PCC of migraineurs without aura, it has several limitations. First, we did not obtain structural information about the precuneus/PCC in migraineurs without aura, and the observed functional abnormalities may be associated with structural abnormalities [35, 36]. In future research, we will combine functional MRI and structural MRI to investigation function and organization in migraineurs without aura. In addition, a previous study reported that female migraineurs had a thicker posterior insula and precuneus cortices compared with male migraineurs and healthy controls of both sexes [37]. Considering the prevalence of migraine, the sample size of migraineurs without aura is relatively small, and our results should be verified using a large sample in a subsequent study. Furthermore, we investigated precuneus/PCC abnormalities in both female and male migraineurs and observed no significant differences in FC and ReHo values between the female and male migraineurs; this results may be due to the limited sample.

## Conclusions

This study shows that migraineurs without aura have increased FC in the precuneus/PCC within the DMN as well as increased ReHo value in the precuneus/PCC. The abnormalities in the precuneus/PCC suggest that migraineurs without aura may exhibit information transfer and multimodal integration dysfunction, which may affect pain sensitivity and processing. Our study highlights precuneus/PCC abnormalities in migraineurs without aura; such abnormalities could play a role in the development of migraine symptoms.

## Abbreviations

BOLD: Blood oxygen level-dependent
DMN: Default mode network
FC: Functional connectivity
fMRI: Functional magnetic resonance imaging
FWE: Family wise error
FWHM: Full width half-maximum
HAMA: Hamilton anxiety scale
HAMD: Hamilton depression rating scale
HIT: Headache impact test
ICA: Independent component analysis
ICHD: International Classification of Headache Disorders
ICs: Independent components
KCC: Kendall’s coefficient of concordance
MIDAS: Migraine disability assessment scale
MNI: Montreal Neurological Institute
MRI: Magnetic resonance imaging
PCA: Principal component analysis
PCC: Posterior cingulate cortex
ReHo: Regional homogeneity
ROI: Region of interest
VAS: Visual analogue scale
